# Complete genome sequence of *Methanoculleus bourgensis* strain MAB1, the syntrophic partner of mesophilic acetate-oxidising bacteria (SAOB)

**DOI:** 10.1186/s40793-016-0199-x

**Published:** 2016-10-12

**Authors:** Shahid Manzoor, Anna Schnürer, Erik Bongcam-Rudloff, Bettina Müller

**Affiliations:** 1 Department of Microbiology, Swedish University of Agricultural Sciences, BioCenter, Uppsala, SE 750 07 Sweden; 2 University of the Punjab, Lahore, Pakistan; 3 Department of Animal Breeding and Genetics Science, Swedish University of Agricultural Science, SLU-Global Bioinformatics Centre, Uppsala, SE 750 07 Sweden

**Keywords:** Syntrophy, Methanogens, Methane production, Biogas process, Syntrophic acetate-oxidising bacteria

## Abstract

*Methanoculleus bourgensis* strain MAB1 has been identified as the hydrogenotrophic partner of mesophilic acetate-oxidising bacteria, a syntrophic relationship operating close to the thermodynamic equilibrium and of considerable importance in ammonia-rich engineered biogas processes. *Methanoculleus bourgensis* strain MAB1 belongs to the order *Methanomicrobiales*, family *Methanomicrobiaceae*, within the phylum *Euryarchaeota*. The genome shows a total size of 2,859,299 bp encoding 3450 predicted protein-encoding genes, of which only 1472 (43 %) have been assigned tentative functions. The genome encodes further 44 tRNA genes and three rRNA genes (5S, 16S and 23S rRNA). This study presents assembling and annotation features as well as genomic traits related to ammonia tolerance and methanogenesis.

**Electronic supplementary material:**

The online version of this article (doi:10.1186/s40793-016-0199-x) contains supplementary material, which is available to authorized users.

## Introduction

In anaerobic digestion processes, syntrophy is a particular important interspecies relationship that is of benefit to all contributing partners and is essential for the methanogenesis of organic matter [1, 2]. Syntrophic interaction operates close to the thermodynamic equilibrium, whereby both partners have to share the limited energy released in the overall reactions [2]. Syntrophic acetate-oxidation (SAO) releases a very small amount of energy (ΔG^o`^ = −35 kJ per mol rct), just enough to support microbial growth. The two-step reaction starts with the oxidation of acetate to CO_2_ and hydrogen/formate performed by so-called syntrophic acetate-oxidising bacteria. This can only proceed when, in a second step, the hydrogen/formate is immediately consumed by a hydrogenotrophic methanogenic archaea reducing CO_2_ to methane, which makes the overall acetate oxidation thermodynamically favourable [1]. In a mesophilic co-culture, the hydrogen partial pressure has been observed to be as low as 1.6–6.8 Pa [3] and in thermophilic co-cultures as low as 10–50 Pa [4].

Hydrogenotrophic methanogens, mainly belonging to the order *Methanomicrobiales* and *Methanobacteriales*, have been shown to be present in high abundances in thermophilic and mesophilic high ammonia biogas digesters [5–8]. *Methanothermobacter* *thermoautrophicus* affiliating to the order Methanobacterales has been isolated as a methanogenic partner in thermophilic SAO [9, 10]. Within the order *Methanomicrobiales*, members of the genus *Methanoculleus* have been reported to be the prevailing species in ammonia-enriched processes dominated by SAO [6, 11–13]. In total, four methanogenic strains have been isolated from ammonia-rich mesophilic biogas processes [11, 14, 15], named MAB1, MAB2, MAB3 and BA1, which are phylogenetically affiliated to the species *Methanoculleus bourgensis*. MAB1 and BA1 have proven to be a suitable methanogenic partner for mesophilic syntrophic acetate-oxidising bacteria *Clostridium ultunense*, “*Tepidanaerobacter acetatoxydans* *”* and *Syntrophaceticus schinkii* [16–18]. One of the major characteristics of SAO communities is that they can tolerate ammonia levels up to 1 g/L, giving them a selective advantage over aceticlastic methanogens, which convert acetate directly to methane and cannot tolerate such high concentrations [6, 8, 11, 19–22].

This study reports the genome sequencing, assembly and annotation of the methanogenic SAOB partner *Methanoculleus bourgensis* strain MAB1, a key organism in methane production from ammonia-rich feed stocks in anaerobic digestion processes.

## Organism information

### Classification and features

**Fig. 1:**
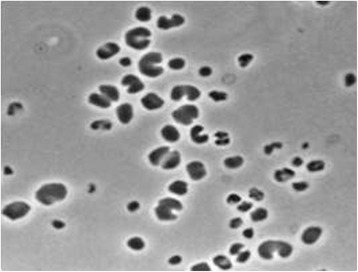
Image. Micrograph of *Methanoculleus bourgensis* strain MAB1

*Methanoculleus bourgensis* MAB1 is an obligate anaerobic archaea that has been isolated from a mesophilic methanogenic reactor operating with swine manure at 6 g NH4 + −N/L and a pH of 7.5. The isolated cells were between 1.0 and 3.0 μm in diameter, irregular and coccoid in shape (Fig. 1) and surrounded by a protein S-layer [11]. The strain forms methane from H_2_/CO_2_, formate, 2-propanol and 1,2-propanol, but not from acetate, which is required for growth. A more detailed description can be found in [11]. Although isolated from mesophilic reactors, the optimal methane production rate has been observed at hyper-mesophilic temperatures of between 44 and 45 °C [23]. It can probably tolerate ammonia concentrations up to 1 g/L [12]. Minimum information about the genome sequence (MIGS) of *M. bourgensis* strain MAB1 is given in Table 1 and Table S1 (Additional file 1).

**Table 1:** Classification and general features *Methanoculleus bourgensis* strain MAB1 according to the MIGS specification [42]

MIGS ID	Property	Term	Evidence code^a^
	Classification	Domain *Archaea*	TAS [43]
		Phylum *Euryarchaeotes*	TAS [44]
		Class *Methanomicrobia*	TAS [45, 46]
		Order *Methanomicrobiales*	TAS [47, 48]
		Family *Methanomicrobiaceae*	TAS [49]
		Genus *Methanoculleus*	TAS [50]
		Species *Methanoculleus bourgensis*	TAS [18, 51]
		Strain *MAB1*	TAS [11]
	Gram stain	Negative	TAS [11]
	Cell shape	Irregular coccus	TAS [11]
	Motility	Not observed	TAS [11]
	Sporulation	Not observed	TAS [11]
	Temperature range	15–50 °C	TAS [23]
	Optimum temperature	44–45 °C	TAS [23]
	Carbon source	CO_2_	TAS [11]
MIGS-6	Habitat	Anaerobic digester	TAS [11]
MIGS-6.3	Salinity	0.0–0.220 M NH_4_Cl	TAS [11]
MIGS-22	Oxygen requirement	Anaerobe	TAS [11]
MIGS-15	Biotic relationship	Syntrophy (beneficial), free living	TAS [11]
MIGS-14	Pathogenicity	Not reported	NAS
MIGS-4	Geographic location	Biogas reactor, Uppsala, Sweden	NAS
MIGS-5	Sample collection	1989	NAS
MIGS-4.1	Latitude	59.8581° N	NAS
MIGS-4.2	Longitude	17.6447° E	NAS
MIGS-4.4	Altitude	not applicable	NAS

^a^Evidence codes - *IDA* Inferred from Direct Assay, *TAS* Traceable Author Statement (i.e., a direct report exists in the literature); *NAS* Non-traceable Author Statement (i.e., not directly observed for the living, isolated sample, but based on a generally accepted property for the species, or anecdotal evidence) These evidence codes are from the Gene Ontology project [52]

**Fig. 2:**
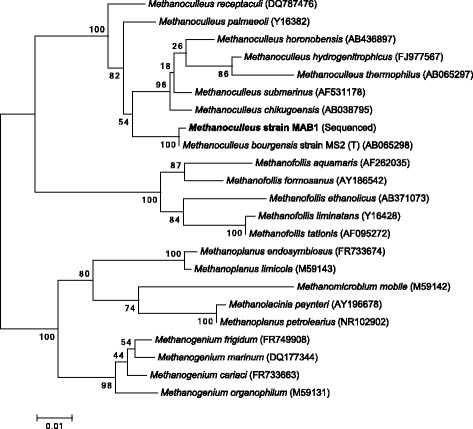
Phylogentic tree. Maximum likelihood tree highlighting the phylogenetic position of *Methanoculleus bourgensis strain* MAB1 within the family *Methanomicrobiacaea.* The 16S rRNA-based alignment was carried out using MUSCLE [53] and the phylogenetic tree was inferred from 1521 aligned characteristics of the 16S rRNA gene sequence using the maximum-likelihood (ML) algorithm [54] with MEGA 6.06 [55, 56]. Bootstrap analysis [57] with 100 replicates was performed to assess the support of the clusters

Phylogenetic analysis of the single 16 s rRNA gene copy affiliates *M. bourgensis* MAB1 to the *Methanomicrobia* class within the phylum *Euryarchaeota* and therein to the family *Methanomicrobiaceae* (RDP Naive Bayesian rRNA Classifier Version 2.10, October 2014). The comparison of the 16S rRNA gene with the latest available databases from GenBank (2016-01-29) using BLAST under default settings have revealed *Methanoculleus marisnigri* JR1 (NC_009051.1) to be the closest current relative, sharing 97 % identity (Fig. 2). The type strain is *Methanoculleus bourgensis* MS2 (T), whose 16 s rRNA gene is 99 % identical to strain MAB1 and which was isolated from a tannery by-product enrichment culture inoculated with sewage sludge [24]. *Methanoculleus olentangyi* and *Methanoculleus oldenburgensis* are subjective synonyms [25]. Cells of *M. bourgensis* strain MAB1 show a polyamine pattern that is distinctly different from the type strain MS2 [11].

## Genome sequencing information

### Genome project history

*Methanoculleus bourgensis* MAB1 was sequenced and annotated by the SLU-Global Bioinformatics Centre at the Swedish University of Agricultural Sciences, Uppsala, Sweden. The genome project is deposited in the Genomes OnLine Database [26] with GOLD id Gb0126792, and the complete genome is deposited in the European Nucleotide Archive database with accession number ERS1044365. This methanogenic partner of SAOB was selected for sequencing on the basis of environmental relevance to issues in global carbon cycling, alternative energy production and geochemical importance. Table 2 contains a summary of the project information.

**Table 2:** Project information

MIGS ID	Property	Term
MIGS-31	Finishing quality	Complete
MIGD-28	Libraries used	Ion Torrent single end reads
MIGS-29	Sequencing platform	Ion Torrent PGM Systems
MIGS-31.2	Sequencing coverage	35×
MIGS-30	Assemblers	Newbler 2.8 and MIRA 4.0
MIGS-32	Gene calling method	PRODIGAL and AMIGene
	Locus Tag	MMAB1
	Genbank ID	LT158599.1
	GenBank Data of release	12-FEB-2016
	GOLD ID	Gb0126792
	BIOPROJECT	PRJEB12532
MIGS 13	Source Material Identifier	Biogas digester sludge
	Project relevance	Biogas production

### Growth conditions and genomic DNA preparation

The strain had been stored as liquid cultures since its isolation in the laboratory. For DNA isolation, batch cultures were grown in basal medium as described by Zehnder et al. [27] and modified by Schnürer et al., [28] supplemented with 5 mM acetate and 0.3 M NH_4_Cl_2_. The headspace was filled with H_2_/CO_2_ (80:20, v/v). Cells were grown over 2 months at 37 °C without shaking, and harvested at 5000 X g. DNA was isolated using the Blood & Tissue Kit from Qiagen (Hilden, Germany) according to the standard protocol, but omitting the lysozyme step. The quality was visualised by agarose gel electrophoresis and the quantity determined by fluorometric measurements using Qubit (Thermo Fisher Scientific, Waltham, MA, USA).

### Genome sequencing and assembly

The genome of *Methanoculleus bourgensis* strain MAB1 was sequenced at the SciLifeLab Uppsala, Sweden using Ion Torrent PM systems with a mean length of 206 bp, a longest read length 392 bp and a total of final library reads of 2,985,963 for single end reads. General aspects about the sequencing performed can be found on the SciLifeLab website [29]. The FastQC software package [30] was used for read quality assessment. After preassembly quality checking, the reads were assembled with MIRA 4.0 and *Newbler* 2.8 assemblers. Possible miss-assemblies were corrected manually using Tablet, a graphical viewer for visualisation of assemblies and read mappings [31]. Whole-genome assembly of the *M. bourgensis* strain MAB1 genome was accomplished using a comparative genome assembly method [32], which combines *de novo* and mapping assemblies. The filtered reads were fed into MIRA version 4.0 [33] for both mapping and *de novo* assembly, and the same read data were also provided to *Newbler* 2.8 *de novo* assembler. Mapping assembly was undertaken against the available genome of *Methanoculleus marisnigri* JR1 (accession no. NC_009051.1). Contigs produced through *de novo* assembly of read data from both assemblers were sorted and oriented along the reference genome and then aligned to the mapping assembly using Mauve genome alignment software [34]. Alignment of contigs to mapping assembly indels covered all the gaps in the genome. These covered gaps were all verified through PCR amplification using a Hot Start High-fidelity DNA polymerase (Phusion, Thermo Fisher Scientific, Waltham, MA, USA) and subsequent Sanger sequencing (Macrogen Corporation, Geumcheon District, South Korea). The complete genome sequence of *Methanoculleus bourgensis* strain MAB1 contained 2,859,299 bp based on the analysis performed using the tools summarised above.

### Genome annotation

Automated gene modelling was completed by MaGe [35], a bacterial genome annotation system. Genes were identified using Prodigal [36] and AMIGene [37] as part of the MaGe genome annotation pipeline. The predicted CDSs were translated and used to search the NCBI non-redundant database and UniProt, TIGRFam, Pfam, PRIAM, KEGG, COG and InterPro databases using BLASTP. Predicted coding sequences were subjected to manual analysis using the MaGe web-based platform, which also provides functional information about proteins and was used to assess and correct genes predicted through the automated pipeline. The predicted functions were also further analysed by the MaGe annotation system (Fig. 4).

## Genome properties

**Fig. 3:**
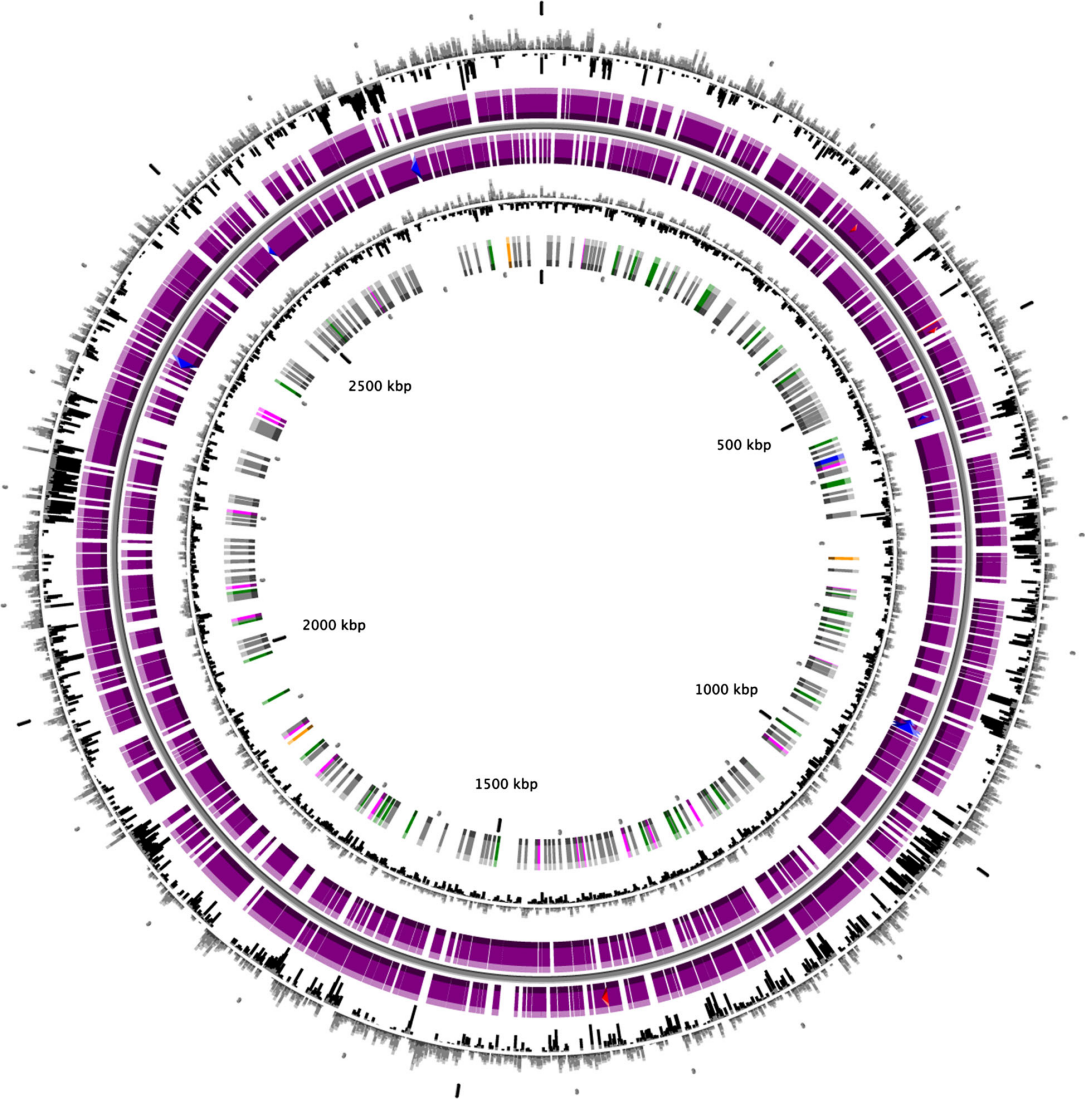
Circular map. Circular map of the *Methanoculleus bourgensis* strain MAB1 genome (from the outside to the centre): (1) GC percent deviation (GC window - mean GC) in a 1000-bp window; (2) predicted CDSs transcribed in a clockwise direction; (3) predicted CDSs transcribed in a counterclockwise direction; (4) GC skew (G + C/G-C) in a 1000-bp window; (5) rRNA (*blue*), tRNA (*green*), misc_RNA (*orange*), transposable elements (*pink*) and pseudogenes (*grey*)

The complete genome comprised a single contig with a total size of 2,859,299 bp and a calculated GC content of 60.26 %. The genome showed a protein coding density of 84.59 % with an average intergenic length of 162.92 bp. The genome encoded a further 44 tRNA genes and three rRNA genes (5S, 16S and 23S rRNA) (Table 3, Fig. 3).

**Table 3:** Genomic statistics

Attribute	Value	% of total
Genome size (bp)	2,859,299	100.00
DNA Coding (bp)	2,430,404	85.00
DNA G + C (bp)	1,723,014	60.26
DNA scaffolds	1	-
Total genes	3507	100.00
Protein coding genes	3450	98.37
RNA genes	44	1.25
Pseudo gene	57	1.62
Genes in internal clusters	1580	45.00
Genes with function prediction	1472	43.00
Genes assigned to COGs	2323	66.24
Genes with Pfam domains	2801	79.84
Genes with signal peptides	332	9.46
Genes with transmembrane helices	650	18.53
CRISPR repeats	3	.08

The genome of *Methanoculleus bourgensis* strain MAB1 genome contained 3450 predicted protein-encoding genes, of which 1472 (43 %) have been assigned tentative functions. The remaining 1978 ORFs were hypothetical/unknown proteins. 2323 (app. 66 %) of all predicted protein-encoding genes could be allocated to the 22 functional COGs. Analysis of COGs revealed that ~21 % of all protein-encoding genes fell into four main categories: energy metabolism (6.4 %), amino acid transport and metabolism (5.9 %), coenzyme transport and metabolism (4.6 %) replication, and recombination and repair (4.2 %) (Table 4).

**Table 4:** Number of genes associated with the general COG functional categories

Code	Value	% age	Description
J	180	5.13	Translation, ribosomal structure and biogenesis
A	2	0.05	RNA processing and modification
K	119	3.39	Transcription
L	146	4.16	Replication, recombination and repair
B	5	0.14	Chromatin structure and dynamics
D	32	0.91	Cell cycle control, cell division, chromosome partitioning
Y	0	0.00	Nuclear structure
V	51	1.45	Defence mechanisms
T	82	2.33	Signal transduction mechanisms
M	114	3.25	Cell wall/membrane/envelope biogenesis
N	30	0.85	Cell motility
Z	0	0.00	Cytoskeleton
W	0	0.00	Extracellular structures
U	29	0.82	Intracellular trafficking, secretion and vesicular transport
O	120	3.42	Posttranslational modification, protein turnover, chaperones
C	224	6.38	Energy production and conversion
G	128	3.64	Carbohydrate transport and metabolism
E	208	5.93	Amino acid transport and metabolism
F	70	1.99	Nucleotide transport and metabolism
H	160	4.56	Coenzyme transport and metabolism
I	42	1.19	Lipid transport and metabolism
P	174	4.96	Inorganic ion transport and metabolism
Q	34	0.96	Secondary metabolites biosynthesis, transport and catabolism
R	429	12.23	General function prediction only
S	333	9.49	Function unknown
	1184	34.00	Not in COGs

## Insights from the genome sequence

**Fig. 4:**
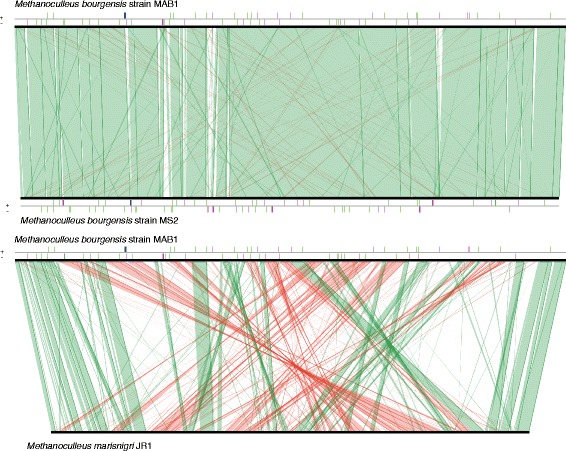
Synteny comparison. Synteny comparison of *Methanoculleus bourgensis* strain MAB1 genome with the closely related genome of *Methanoculleus marisnigri* strain JR1 and the type strain *Methanoculleus bourgensis* strain MS2. Linear comparisons of all predicted gene loci from *Methanoculleus bourgensis* strain MAB1 with *Methanoculleus marisnigri* strain JR1 and *Methanoculleus bourgensis* strain MS2, respectively, were performed using the built-in tool in MaGe platform with the synton size of > = three genes. The lines indicate syntons between two genomes. *Red lines* show inversions around the origin of replication. *Vertical bars* on the border line indicate different elements in genomes such as *pink*: transposases or insertion sequences: *blue*: rRNA and *green*: tRNA

Synteny-based analysis revealed that *Methanoculleus bourgensis* strain MAB1 had approximately 55 % of the total genome size in synteny with its closest relative *Methanoculleus marisnigri* JR1 (Fig. 4)*.* The type strain *Methanoculleus bourgensis* strain MS2 had approximately 70 % of the total genome size in synteny with *Methanoculleus bourgensis* strain MAB1 (Fig. 4). A comparison of all inferred proteins of *M. bourgensis* strain MAB1 with all proteins collected in the NCBI RefSeq database revealed the highest number of orthologous (2800: 79.75 %) with *M. bourgensis* strain MS2 and next to *M. marisnigri* JR1 (2163: 61.61 %).

Analysis of COGs revealed that 2323 (app. 66 %) of all predicted protein-encoding genes of *M. bourgensis* *strain MAB1* could be allocated to the 22 functional COGs, which is slightly lower than that predicted for *M. bourgensis* strain MS2 (2072 genes; 69 %) and for *M. marisnigri* JR1 (2016 genes; 75 %), where the protein-encoding genes of both could be allocated to 23 functional GOGs.

Although *Methanoculleus bourgensis* strain MAB1 has not yet been observed to express a flagellum, a cluster (MMAB1_2416, MMAB1_2434;MMAB1_0328) encoding flagellum (*flaB,H,J,K*) and chemotaxis (*cheW,A,D,C*) related genes (MMAB1_2416-2434) indicate chemotactic capabilities [38].

The genome does not contain genes related to ammonium transport systems, which have been found to be encoded by the genome of its close relative *Methanoculleus marisnigri* (BlastP search using ammonium transporter of *M. marisnigri* as protein query), and might therefore be considered an adaptation to high osmolarity environments and to high ammonium levels in particular. The same genotype has also described for the type strain MS2 [39] and for its acetate-oxidising syntrophic partner organism “*T. acetatoxydans* *”* [40] and *S. schinkii* *(unpublished)*. As also predicted for the type strain MS2 [39], a putative potassium ABC transport system (MMBA1_2581,MMAB1_2585) with an adjacent two-component regulatory system (MMAB1_2586, MMAB1_2587), two cation transporter (MMAB1_0409, MMAB1_1566), two potassium antiporter (MMBA1_1794, MMAB1_2374), a choline/carnitine/betaine transporter and one glycine/betaine ABC transport system might be involved in osmoregulation.

The genes encoding the methanogenesis pathway from H_2_ and CO_2_, including formylmethanofuran dehydrogenase (MMAB1_2225-MMAB1_2227), formylmethanofuran-tetrahydromethanopterin fromyltransferase (MMAB1_2217), methenyltetrahydromethanopterin cyclohydrolase (MMAB1_2292), methylenetetrahydromethanopterin dehydrogenase (MMAB1_2159), methylenetetrahydromethanopterin reductase (MMAB1_2155), tetrahydromethanopterin S-methyltransferase (MMAB1_2236-MMAB1_2244), methyl-CoM reductase (MMAB1_2231-2235) and CoB-CoM heterodisulfide reductase (MMAB1_2220), were found clustering together. The genome further revealed duplicates in the case of formylmethanofuran dehydrogenase (MMAB1_1584-1586, MMAB1_1958-1962) and methyl-CoM reductase (MMAB1_1952-MMAB1_1956). The expression levels of the two methyl-CoM reductases encoded by the genome of *M. thermoautotrophicus* were shown to be dependent on syntrophic or autotrophic growth conditions [41].

Moreover, the genome of *M. bourgensis* strain MAB1 codes for three formate dehydrogenases (MMBA1_1689-1690, MMAB1_1105-1106, MMAB1_2913-2914), as has also been described for strain MS2 [39] as well as a putative formate/nitrite transporter (MMAB1_1101/2). These proteins might be involved in efficient uptake and utilisation of formate by donating electrons from formate via formate dehydrogenases to the heterodisulfide reductase, an electron-bifurcating mechanism recently suggested for *Methanococcus maripaludis* [40].

## Conclusions

*Methanoculleus bourgensis* strain MAB1 has been identified as a syntrophic partner for acetate-oxidising bacteria in biogas processes operating with high ammonia levels. Initial genome surveillance indicates an adaption of strain MAB1 to the high osmolarity of this particular environment, as has also been observed for its syntrophic partner organisms. It reveals further gene sets likely to mediate efficient formate uptake and conversion, a possible end product of acetate oxidation. There is a remarkable discrepancy between *Methanoculleus bourgensis* strain MAB1 and the type strain *Methanoculleus bourgensis* strain MS2, as indicated by the number of orthologous and synteny percentages. Follow-up genome analysis and –omics approaches will investigate these differences further and elucidate what is specific about strain MAB1 that makes it the preferred partner organism of this particular syntrophy.

## Additional file

Additional file 1: Table S1. Associated MIGS record. (DOCX 124 kb)

## Abbreviations

SAOB: syntrophic acetate-oxidising bacteria
MIRA: Mimicking Intelligent Read Assembly, MaGe, Magnifying Genomes
BLASTP: Basic local alignment search tool for proteins
NCBI: National Center for Biotechnology Information
