# Supplementary material for: Complete genome sequence of Methanoculleus bourgensis strain MAB1, the syntrophic partner of mesophilic acetate-oxidising bacteria (SAOB)
Source: Stand Genomic Sci. 2016 Oct 12;11:80. doi: 10.1186/s40793-016-0199-x (PMC5062929; doi:10.1186/s40793-016-0199-x)
Supplement: Additional file 1: Table S1. — Associated MIGS record. (DOCX 124 kb) [file 40793_2016_199_MOESM1_ESM.docx]

**Associated MIGS Record**

**Table S1.** Associated MIGS record.

| **MIGS-ID** | field name | description |
| --- | --- | --- |
| **MIGS-1** | Submit to INSDC/Trace archives |  |
| **1.1** | PID | [PRJEB12532](http://www.ncbi.nlm.nih.gov/bioproject/PRJEB12532) |
| **1.2** | Trace Archive | ERS1044365 |
| **MIGS-2** | MIGS CHECK LIST TYPE |  |
| **MIGS-3** | Project Name | Complete genome sequence of [Methanoculleus bourgensis](http://dx.doi.org/10.1601/nm.179) strain MAB1, the syntrophic partner of mesophilic acetate-oxidising bacteria (SAOB) |
| **MIGS-4** | Geographic Location | Sweden |
| **4.1** | Latitude | 59,849998 |
| **4.2** | Longitude | 17,63333 |
| **4.3** | Depth | Not reported |
| **4.4** | Altitude | Not reported |
| **MIGS-5** | Time of Sample collection | 1989 |
| **MIGS-6** | Habitat (EnvO) | Anaerobic sludge |
| **6.1** | temperature | Mesophilic |
| **6.2** | pH | 7.5 |
| **6.3** | salinity | Not reported |
| **6.4** | chlorophyll | Not reported |
| **6.5** | conductivity | Not reported |
|  |  |  |
| **6.6** | light intensity | Not reported |
| **6.7** | dissolved organic carbon (DOC) | Not reported |
| **6.8** | current | Not reported |
| **6.9** | atmospheric data | Not reported |
| **6.10** | density | Not reported |
| **6.11** | alkalinity | Not reported |
| **6.12** | dissolved oxygen | Not reported |
| **6.13** | particulate organic carbon (POC) | Not reported |
| **6.14** | phosphate | Not reported |
| **6.15** | nitrate | Not reported |
| **6.16** | sulfates | Not reported |
| **6.17** | sulfides | Not reported |
| **6.18** | primary production | Not reported |
| **MIGS-7** | Subspecific genetic lineage |  |
| **MIGS-9** | Number of replicons | 01 |
| **MIGS-10** | Extrachromosomal elements | None |
| **MIGS-11** | Estimated Size | 2.9 Mbp |
| **MIGS-12** | Reference for biomaterial or Genome report |  |
| **MIGS-13** | Source material identifiers |  |
| **MIGS-14** | Known Pathogenicity | None |
|  |  |  |
| **MIGS-15** | Biotic Relationship | Syntrophy |
| **MIGS-16** | Specific Host | None |
| **MIGS-17** | Host specificity or range (taxid) |  |
| **MIGS-18** | Health status of Host |  |
| **MIGS-19** | Trophic Level |  |
| **MIGS-22** | Relationship to Oxygen | Obligate anaerobe |
| **MIGS-23** | Isolation and Growth conditions | H_2_/CO_2_, 5 mM acetate |
| **MIGS-27** | Nucleic acid preparation | Blood&Tissue kit, Qiagen |
| **MIGS-28** | Library construction |  |
| **28.1** | Library size | 206 bp |
| **28.2** | Number of reads | 2,985,963 |
| **28.3** | vector |  |
| **MIGS-29** | Sequencing method | Ion Torrent |
| **MIGS-30** | Assembly | MMAB1 |
| **30.1** | Assembly method | *De novo*, Mapping |
| **30.2** | estimated error rate |  |
| **30.3** | method of calculation |  |
| **MIGS-31** | Finishing strategy |  |
| **31.1** | Status | Complete |
| **31.2** | coverage | 35x |
| **31.3** | contigs | 01 |
| **MIGS-32** | Relevant SOPs |  |
| **MIGS-33** | Relevant e-resources |  |
